# Learned Primal Dual Reconstruction for PET

**DOI:** 10.3390/jimaging7120248

**Published:** 2021-11-24

**Authors:** Alessandro Guazzo, Massimiliano Colarieti-Tosti

**Affiliations:** 1 Division of Biomedical Imaging, KTH Royal Institute of Technology, 10044 Stockholm, Sweden; alessandro.guazzo95@gmail.com; 2 Department of Clinical Science, Intervention and Technology, Karolinska Institutet, 17177 Stockholm, Sweden

**Keywords:** PET, tomographic reconstruction, inverse problems, deep learning

## Abstract

We have adapted, implemented and trained the Learned Primal Dual algorithm suggested by Adler and Öktem and evaluated its performance in reconstructing projection data from our PET scanner. Learned Primal Dual reconstructions are compared to Maximum Likelihood Expectation Maximisation (MLEM) reconstructions. Different strategies for training are also compared. Whenever the noise level of the data to reconstruct is sufficiently represented in the training set, the Learned Primal Dual algorithm performs well on the recovery of the activity concentrations and on noise reduction as compared to MLEM. The algorithm is also shown to be robust against the appearance of artefacts, even when the images that are to be reconstructed present features were not present in the training set. Once trained, the algorithm reconstructs images in few seconds or less.

## 1. Introduction

Positron Emission Tomography (PET) is an inherently three-dimensional molecular imaging technique that is able to image the distribution of an injected radioactive tracer *in vivo* (for an introduction see, for example, [1,2]). PET image reconstruction deals with the tomographic inverse problem of finding an image, f∈X, given a finite number of noisy projections, g∈Y. Measured PET projection data, *g*, can be seen as a noisy realisation of a PET forward-projection operator, A:X→Y, that acts on the 3D activity map, *f*. Here, A models how an activity map gives rise to PET data and includes the data-acquisition geometry, which is mainly dictated by the arrangement of PET detectors. The PET image-reconstruction problem can then be seen as the task of estimating the unknown 3D activity map, *f*, from data, g=A(f)+ν, where ν is generated by a random variable and represents observation error and quantum noise.

Ionising-radiation based medical imaging faces a compelling trade-off between image quality and dose, and this, together with the relatively low detection efficiency of PET, pushes data acquisition towards a low-count regime, where Poisson noise dominates. Nowadays, the standard clinical reconstruction method for PET images is Maximum Likelihood Expectation Maximisation (MLEM) [3,4,5] or its equivalent, computationally more efficient, Ordered Subset Expectation Maximisation (OSEM) [6]. MLEM has its strength in the correct modelling of the Poisson noise that dominates PET-projections and images, while its main drawback is in its slow convergence rate and in a high variance at high iteration numbers [7]. In clinical practice, MLEM reconstructions are regularised by heuristically determined early stopping in the iteration process [8].

The most successful attempts beyond MLEM have been algorithms in which regularisation is obtained through the action of a prior. Notable examples are the Maximum *a Posteriori* (MAP) formulation, where one seeks to maximise the likelihood of an image given the data and an *a priori* probability distribution of the images, and variational algorithms based on the solution of the optimisation problem:

(1) $$\underset{f}{\mathrm{argmin}}∥Af-g∥+\lambda P(f),$$

where λ is a positive constant, ∥·∥ indicates a distance, and P(·) typically encodes some prior knowledge on *f* that is used for regularisation. λ is a hyperparameter of the optimisation and has to be determined in some way. This fact, together with the computational heaviness of this family of reconstruction algorithms, are the main drawbacks of this family of algorithms. In the last few years there have been attempts of making use of (deep) Convolutional Neural Networks (CNNs) in combination with one of the methods previously described. Leaving out of this introduction simple denoising, and without attempting at giving an exhaustive review of the literature, most of the efforts can be divided in two categories:

- Methods using CNNs to enforce a learned penalty in a Maximum a Posteriori method, such as the Maximum a Posteriori Expectation Maximisation (MAP EM) net [9].
- Methods in which CNNs are used as a way of learning an image parametrisation, such as the Convolutional Neural Network Representation [10].

Notably, in both of the cases above, CNNs are only used to map images to images. Adler and Öktem [11,12] proposed instead to use (Deep) Neural Networks both in data and in image space. This approach is particularly interesting in PET imaging, where a substantial part of the effort to obtain better images is spent in data correction (e.g., sensitivity normalisation, scatter and random correction) prior to reconstruction.

In this paper, we present the results we obtained applying a modified version of the Learned Primal Dual (LPD) algorithm [12] to data obtained from miniPET-3 [13,14], an in-house developed PET-scanner dedicated to small animal imaging. We also show that a hybrid training approach that mixes rather extensive training on synthetic data and on a limited amount of experimental data is a promising method for making deep-learning aided algorithms like the LPD clinically viable.

## 2. Materials and Methods

### 2.1. Theoretical Background

This paper focuses on the results obtained with the Learned Primal Dual (LPD) algorithm by Adler and Öktem [12] applied to PET tomographic reconstruction. We now briefly present the algorithm.

Let us consider images *f* in image space X and data g∈Y, a forward operator, A, that maps, to some approximation, images to data: A:X→Y:Af≈g, and its adjoint, $A^{*}$. Let us also define a family of operators, $\Lambda_{\theta_{i}}^{i}:\overset{(i+1)-\mathrm{times}}{\overset{︷}{X\times X....\times X}}\to X$, with parameters $\theta_{i}$ and a corresponding family of operators in data space, $\Xi_{\phi_{i}}^{i}:\overset{(i+1)-\mathrm{times}}{\overset{︷}{Y\times Y....\times Y}}\to Y$, with parameters $\phi_{i}$. The index *i* here tracks the number of LPD steps in the reconstruction algorithm (see Algorithm 1 in the following). The main idea of the LPD algorithm is to use the forward operator A and its adjoint $A^{*}$ for modelling, respectively, the image to data and the data to image mapping, while convolutional neural networks (CNN) are used for image to image and data to data updates. Their role is to enforce learned priors on images and data.

**Figure 1:**
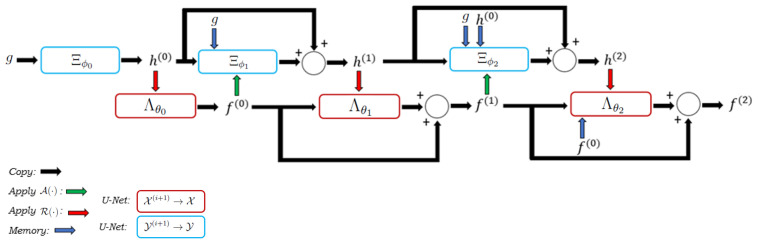
A graphical representation of the LPD algorithm in the three-step case is shown.

The full LPD algorithm is:
**Algorithm 1** Learned Primal-Dual reconstruction.1:Given: *g*, $N_{\mathrm{LPD}}$2:Set: $R\left(\cdot \right)=\frac{A^{*}\left(\cdot \right)}{{\left||A|\right|}^{2}}$3:$h^{\left(0\right)}=\Xi_{\phi }^{0}\left(g\right)$4:$f_{g}^{\left(0\right)}=R\left(h^{\left(0\right)}\right)$5:$f^{\left(0\right)}=\Lambda_{\theta }^{0}\left(f_{g}^{\left(0\right)}\right)$6:**for**i=1, ⋯, $N_{\mathrm{LPD}}-1$**do**7:    $h_{f}^{\left(i-1\right)}=A\left(f^{\left(i-1\right)}\right)$8:    $h^{\left(i\right)}=h^{\left(i-1\right)}+\Xi_{\phi }^{i}\left(g,h^{\left(0\right)},\cdots ,h^{\left(i-1\right)},h_{f}^{\left(i-1\right)}\right)$9:    $f_{g}^{\left(i\right)}=R\left(h^{\left(i\right)}\right)$10:    $f^{\left(i\right)}=f^{\left(i-1\right)}+\Lambda_{\theta }^{i}\left(f^{\left(0\right)},\cdots ,f^{\left(i-1\right)},f_{g}^{\left(i\right)}\right)$11:**end for**12:**return**$f^{\left(i\right)}$and is also depicted in Figure 1 for the three iteration case ($N_{\mathrm{LPD}}=3$). Please, note that, at each step, all previous image- and data-iterates are fed to the networks.

### 2.2. Forward Operator and Convolutional Neural Networks Architecture

**Figure 2:**
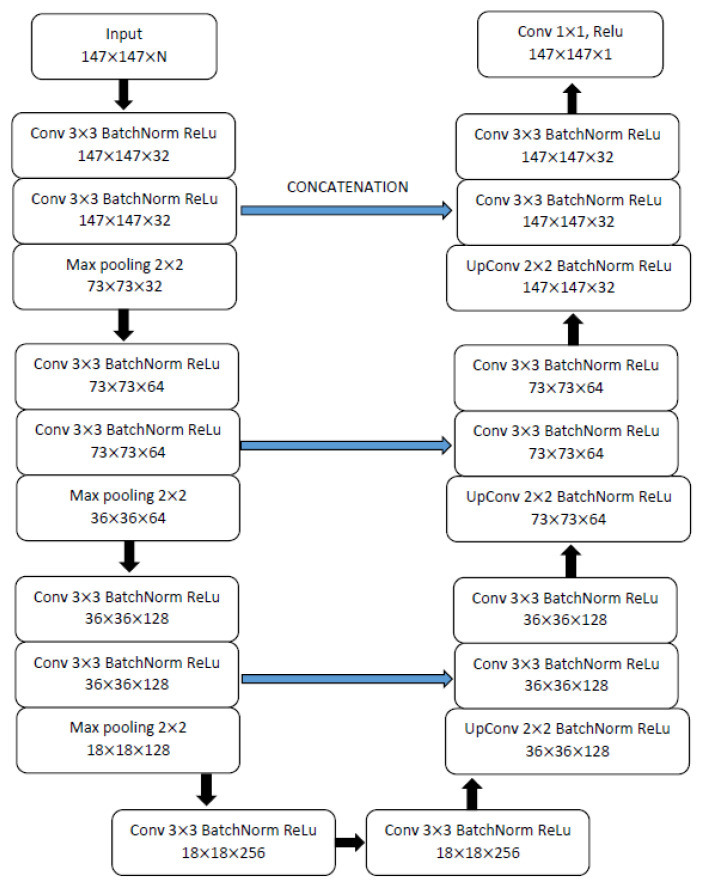
Architecture of the U-Net used at each single step of the LPD-algorithm. The first input has dimensions 147 · 147 · N, with N being equal to the iteration or step number, since the present, as well as all previous iterates, are fed into the CNN at each step.

The forward operator, A, and its adjoint, $A^{*}$, are computed with STIR [15]. MiniPET-3 [13,14] is modelled as a cylindrical scanner with 12 detector modules, each with 35 × 35 crystals. Only the simplest version of the forward operator (i. e., the Radon transform) is used. Images are reconstructed in a 147 × 147 × 1 space, using STIR:s zoom function to reduce the size of the problem while maintaining full system resolution. $\Lambda_{\theta_{i}}^{i}$ and $\Xi_{\theta_{i}}^{i}$ are represented by CNNs implemented in PyTorch (https://pytorch.org, version 0.1.7, accessed on 2 February 2017). They have a 3-layer U-Net architecture described in Figure 2, with 2,143,329 parameters. Python notebooks with the full implementation are available at https://github.com/AlessandroGuazzo/Deep-Learning-for-PET-Imaging/tree/master/miniPET (accessed on 5 March 2021). The implementation makes large use of the utilities of the Operator Discretization Library (ODL) [16].

### 2.3. Training Data Sets and Strategy

#### 2.3.1. Synthetic Data Training

**Figure 3:**
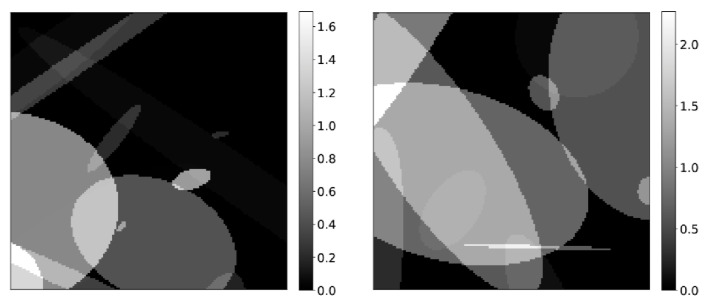
Example of synthetic images used for generating training data.

Training of each of the CNNs in the LPD-algorithm has been achieved mostly via synthetic data. Objects consisting of randomly distributed ellipsoids, with randomly assigned activity concentrations have been generated (see Figure 3 for an example) and corresponding data has been created by applying the forward operator described in Section 2.2. Poisson noise has been added to the data by element-wise dividing the sinograms by a constant value between 3 and 10, and then sampling from a Poisson distribution with mean equal to the resulting pixel value. Appropriate values are then recovered by multiplying again for the relative constant.

In all cases, the Adam optimisation algorithm [17] has been used as optimiser and the smooth L${}^{1}$ distance as loss function.

Training started by training the $\Xi_{\phi_{0}}^{0}$ with $10^{5}$ noisy and noise-free sinograms in a single epoch and with a batch size of 5 and a learning rate of $1.5\cdot 10^{-3}$. The first iteration of the LPD-algorithm has been trained using as a starting point the parameters obtained at the previous training step for the $\Xi_{\phi_{0}}^{0}$ network and the parameters obtained from the training of a simple U-Net denoiser for the $\Lambda_{\theta_{0}}^{0}$ network. Training is then performed end-to-end with $3.75\cdot 10^{5}$ training couples in three epochs with a batch size of 10 and a learning rate of $1.5\cdot 10^{-3}$. The remaining iterations of the LPD-algorithms ($N_{\mathrm{LPD}}=2,3$) have been then added one at the time and the training has been repeated end-to-end, each time, initialising the networks with the parameters obtained in the previous training session, but for the first layers of each CNN, which were initialised with Xavier initialisation [18]. The second and third LPD-iterations were trained with $3.75\cdot 10^{5}$ objects in 3 epochs, a batch size of 5 and a learning rate of $1.5\cdot 10^{-3}$. Finally, the entire 3-iteration LPD-algorithm was trained end-to-end with sets of $6\cdot 10^{5}$ training data in 3 epochs with batch size 5 and learning rate of $1.5\cdot 10^{-3}$ until the loss function had reached acceptable values.

#### 2.3.2. miniPET-3 Data Training

In order to improve the performance of the LPD-algorithm on experimental data, the training from synthetic data has been augmented by also using data obtained from our miniPET-3 scanner. We describe here the procedure for this training step.

**Figure 4:**
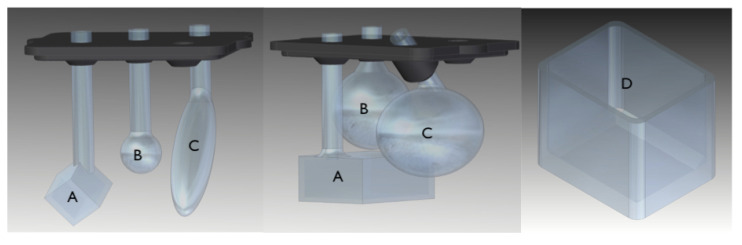
Training phantom #1 and #2. The insert on the left plus the container on the right constitute phantom #1. The insert in the centre in the container on the right is phantom #2. Objects in the inserts and the container are filled with activity (FDG) in concentrations specified in Table 2.

**Figure 5:**
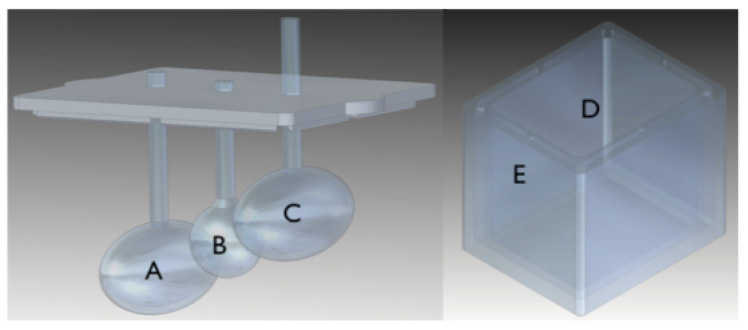
Training phantom #3. Insert on the left, container on the right.

We designed and 3D-printed three different training phantoms shown in Figure 4 and Figure 5. The smallest feature in the training phantoms is the sphere (labelled B) of phantom #1 (see Figure 4), with a total volume of circa 0.2 mL and a diameter of around 8 mm. The largest feature is the ellipsoid (labelled C) of phantom #2 (also shown in Figure 4), with a volume of circa 6 mL.

Training data was generated by measuring, for each experimental configuration described in Table 1, 60 one-minute-equivalent long acquisitions with a coincidences time window of 3 ns and an energy-discrimination window between 350 keV and 650 keV. Coincidences were corrected for random events with a time-delay strategy and normalisation was applied to data before generating sinograms [13]. Please, note that no scatter correction was applied at any stage.

**Figure 6:**
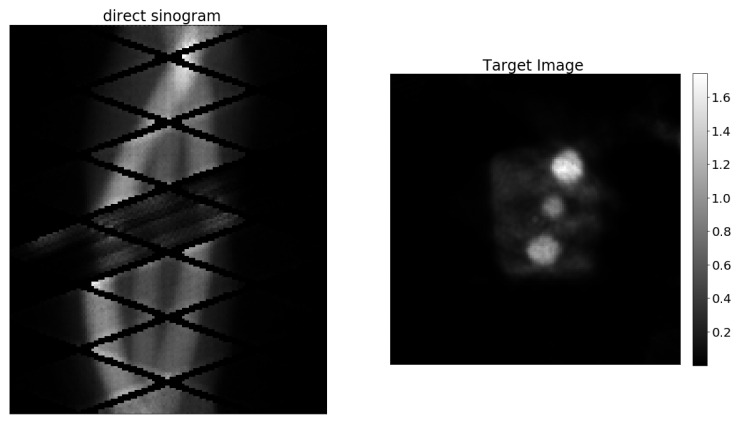
Example of experimental training data from miniPET-3.

In all cases, acquisition times were corrected for giving a one-minute equivalent of data by increasing the actual acquisition time according to the half-life of F18. From each measurement, 60 different noise levels are obtained by adding, one at the time, data from the 60 one minute acquisitions. For each measurement, the 60 direct sinograms obtained after the data-processing step are coupled with the same target image obtained from the full data set. The target image is obtained using the miniPET-3 reconstruction software that performs a 20 iteration MLEM reconstruction. Figure 6 shows a miniPET-3 training data set couple.

Experimental data has then been used for creating two different training sets that are going to be referred to as miniPET data only and hybrid data, and are described in the following:

- miniPET data only: Only miniPET data are inserted in the training set, the training set size is thus 35,700 pairs, since we created 60 different noise levels for each measurement and from each 3D volume we can extract 35 two dimensional slices.
- Hybrid training: In this case, a mix of miniPET data and synthetic data are inserted in the training set. The number of synthetic data is fixed equal to a quarter of the miniPET data set size. The total hybrid training set size is thus 44,625.

Training is then performed, in both cases, in 10 epochs with batch size equal to 15 and learning rate of $1.5\cdot 10^{-3}$ using Adam as optimiser and the smooth L${}^{1}$ distance as loss function. After each epoch, the performance of the trained networks is evaluated on the miniPET-3 test data subset, using the same loss used for the training and the parameters of the model are saved. The model parameters that lead to the smallest loss on the miniPET test data set are chosen as the final ones.

### 2.4. Test Data Set

**Figure 7:**
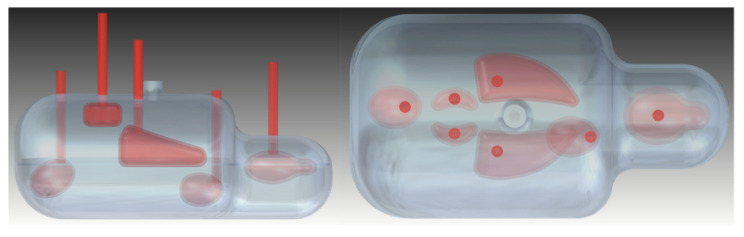
Mouse-like test phantom. Side view (**left**) and top view (**right**).

In order to test the performance of the LPD-algorithm, a mouse-like test phantom has been designed and 3D-printed (see Figure 7). This test phantom was loaded with activity concentrations described in Table 2 and, also in this case, 60 one-minute-equivalent long acquisitions were measured. Random correction, normalisation and sinogram generation were performed in the same way as for data from training-phantom measurements. Additionally, the generation of the test data set and of the target image followed the same strategy as for the training set.

#### Performances Evaluation

Results are evaluated by visual inspection and considering the recovery of the correct activity concentration as a key factor. We have also evaluated the Peak Signal to Noise Ratio (PSNR) and the Structural Similarity index (SSIM). The PSNR has been calculated as follows:

(2) $$\mathrm{PSNR}=10\log_{10}\left(\frac{\max^{2}\left(I\right)}{\mathrm{MSE}}\right)$$

(3) $$\mathrm{MSE}=\frac{1}{mn}\sum_{i=0}^{m-1}\sum_{j=0}^{n-1}{\left[I\left(i,j\right)-K\left(i,j\right)\right]}^{2}$$

where:

- *m*: number of rows of the image,
- *n*: number of columns of the image,
- *I*: noise-free version of the image,
- *K*: noisy image,
- max(I): maximum value of the noise-free version of the image.

The SSIM has been estimated according to:

(4) $$\mathrm{SSIM}=\frac{\left(2\mu_{x}\mu_{y}+C_{1}\right)\left(2\sigma_{xy}+C_{2}\right)}{\left(\mu_{x}^{2}+\mu_{y}^{2}+C_{1}\right)\left(\sigma_{x}^{2}+\sigma_{y}^{2}+C_{2}\right)}$$

where:

- $\mu_{x}$: average value of image *X*,
- $\mu_{y}$: average value of image *Y*,
- $\sigma_{x}^{2}$: variance of image *X*,
- $\sigma_{y}^{2}$: variance of image *Y*,
- $\sigma_{xy}$: covariance of images *X* and *Y*,
- $C_{1}$ and $C_{2}$: constants to avoid computational issues when $\left(\mu_{x}^{2}+\mu_{y}^{2}+C_{1}\right)$ or $\left(\sigma_{x}^{2}+\sigma_{y}^{2}+C_{2}\right)$ is close to zero.

For synthetic data, both figures of merit are computed as the average of the values obtained on 77 different slices of the Shepp–Logan phantom with different levels of noise. For miniPET-3 data both figures of merit are instead computed as the average of the values obtained on the 35 slices of the 3D volume.

## 3. Results

### 3.1. Results on Synthetic Data

This section presents our results on synthetic data, obtained with the LPD-algorithm trained only on synthetic data.

As described in Section 2.3.1, synthetic data for evaluation were generated by forward projection on the Shepp–Logan phantom. Noise of different levels was then added to the projections.

**Figure 8:**
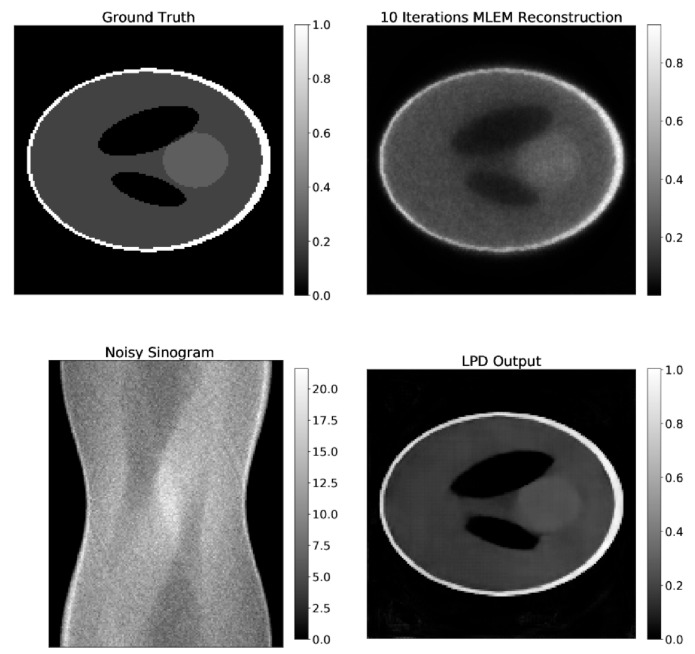
Comparison of LPD-reconstruction and 10-iterations MLEM on synthetic data. The ground truth image and the projection data are also shown.

Examples of the result of the LPD-three iteration algorithm and 10-iteration MLEM, applied on the same data are shown in Figure 8. Additionally, the ground truth image and the corresponding data are shown in the same figure.

For a quantitative comparison, values of SSIM and PSNR for LPD and MLEM are reported in Table 3. These values are the average value over the entire test set, where different levels of noise are added on the data obtained by forward projection. We report also the increase in these two figures of merit (ΔPSNR and ΔSSIM) compared to the 10-iteration MLEM reconstruction on the same data set.

From visual inspection of the reconstructions and from the PSNR and SSIM values, one can conclude that the LPD-algorithm well recovers the activity concentrations of the phantom and scores comparably to MLEM on both PSNR and SSIM. None of the algorithms can recover the smaller ellipsoids of the Shepp–Logan, since those are under the spatial resolution of the system.

### 3.2. Results on miniPET-3 Data

In this section, we present results obtained with LPD-algorithm applied on data from our miniPET-3 scanner. We show results obtained with training on synthetic data only, on a mixture of synthetic and experimental data (hybrid training) and on experimental data only (miniPET data only).

**Figure 9:**
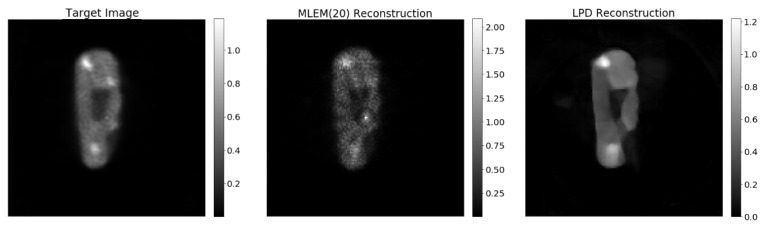
The target image is the 20-iteeration reconstruction on the full data set. The MLEM(20) reconstruction is obtained with only 68% of the data just like the LPD 3 iterations.

In Figure 9, we compare the reconstruction by the LPD-algorithm with hybrid training with what is obtained with 20-iteration MLEM on the same data set (68% of the full data). Due to the lack of a ground truth, the 20-iteration MLEM reconstruction from the full data set is used as target image and considered the benchmark for any comparison.

**Figure 10:**
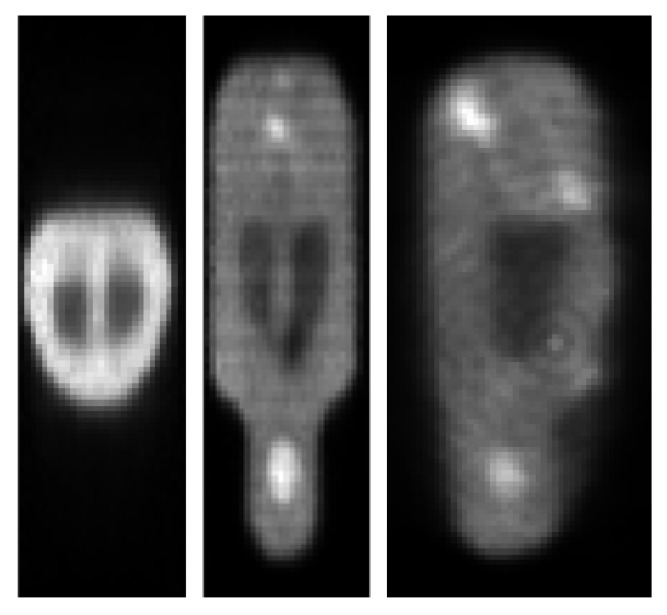
Target image reconstruction of the mouse-like phantom from the full projection data set; transverse, coronal and sagittal planes.

**Figure 11:**
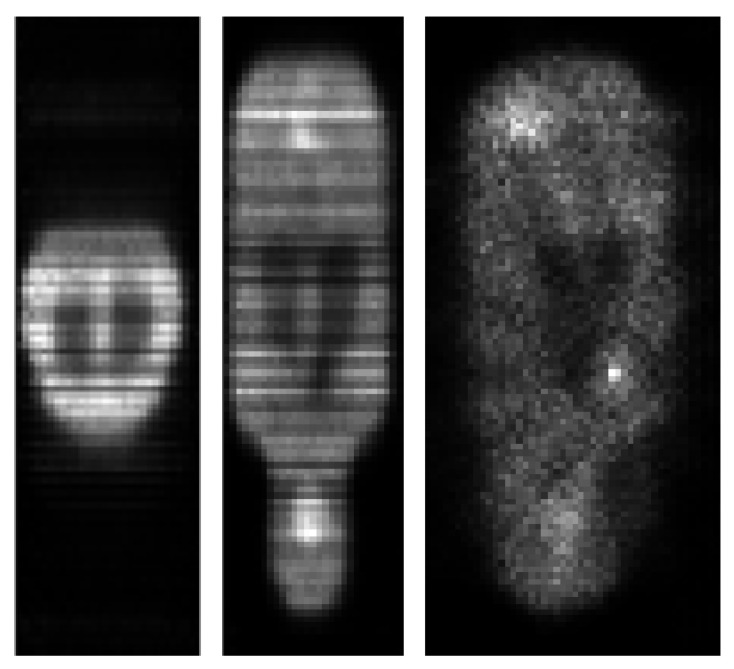
${\mathrm{MLEM}}_{20}$ reconstruction of the mouse-like phantom from 68% of the full projection data set; transverse, coronal and sagittal planes.

**Figure 12:**
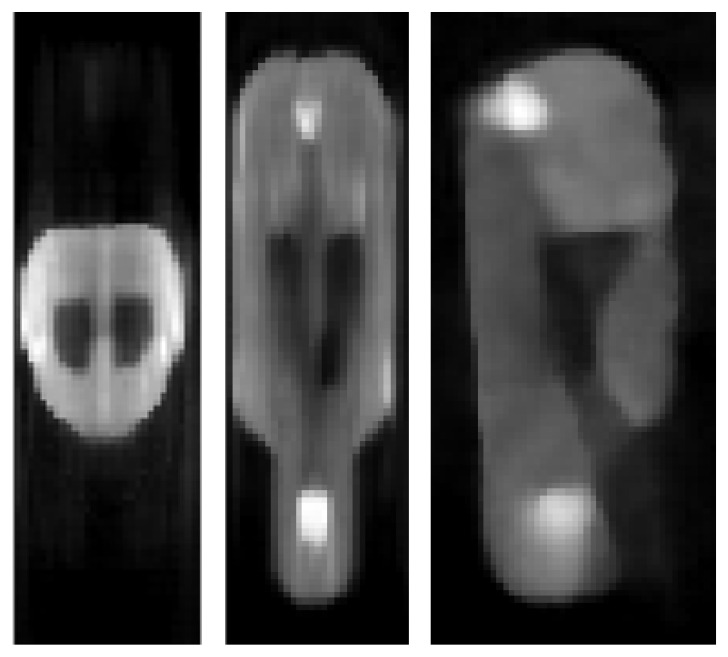
LPD reconstruction of the mouse-like phantom from 68% of the full projection data set; transverse, coronal and sagittal planes.

In Figure 10, Figure 11 and Figure 12, we show reconstructed images from different views.

A comparison of the performance of the synthetic-data-only, hydrid and miniPET-data-only training, is shown in Figure 9.

Table 4 shows the PSNR and SSIM, as well as the gain against 20-iterations MLEM on the same data (68% of the total) evaluated with the help of the target image (100% data, 20-iteration MLEM).

The 3-iteration LPD-algorithm, trained with hybrid data, gives acceptable results, which at visual inspection seem to outperform MLEM reconstructions. In particular, the image obtained with LPD is smoother, less noisy and shows better contrast of the cold areas against the background. The artefact near the top of the lung-like volume is also eliminated. PSNR values tend to confirm this impression, while SSIM favours the 20-iteration MLEM image (see Table 4).

## 4. Discussion

**Figure 13:**
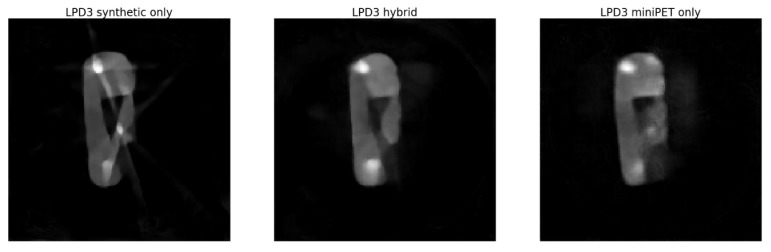
Reconstruction with the 3-iteration LDP-algorithm trained with: synthetic data only (**left**), hybrid training (**centre**) and miniPET only training (**right**).

The main result of our investigation is, in our opinion, that the LPD-algorithm is capable of performing relatively well even when trained with a quite small training set of experimental data, provided that a sufficient training on synthetic data is also performed. Both visual inspection and quantitative analysis, via PSNR, SSIM or the recovered activity concentrations, show acceptable agreement with the target reconstruction using a fraction of the data. Comparing the reconstruction by LPD-algorithm trained only on miniPET data of Figure 13 (right pane) with the target image, one could speculate that extensive training on miniPET data only would possibly give better results than the ones achieved with hybrid training. While this is almost obvious, it is also obvious that such training would be cumbersome, time consuming and, in practice, unfeasible. The hybrid training is, therefore, deemed to be the best viable approach.

**Figure 14:**
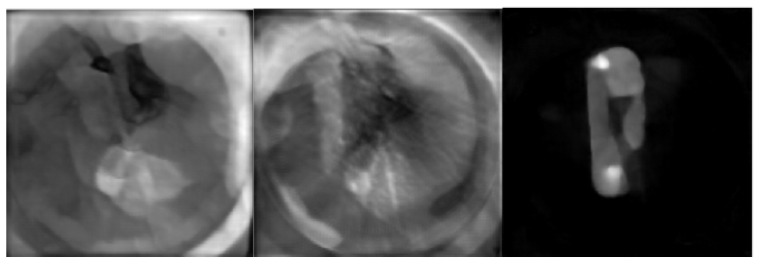
Output of the first (**left**), second (**centre**) and third (**right**) iteration of the LPD-algorithm.

Another interesting outcome of our study has been the observation of how the LPD-algorithm performs a reconstruction with increasing iterations. In fact, the term iteration is probably misleading, since the algorithm does not proceed in steps that come closer and closer to the final solution, but rather focuses on different tasks in each of its three branches. This is shown in Figure 14, where the output of the algorithm at each step is shown. This behaviour is triggered by the fact that all previous iterates, as well as the present, are fed to the CNNs in order for them to output the next iterate. Our intention with this was to make sure that the information contained in the projection data was always contributing to the final output, ensuring that the final result would not be incompatible with the measured data. A careful study of the behaviour of the algorithm at each step could probably lead to a more efficient design of the networks involved in the algorithm, and we intend to investigate this in the future.

**Figure 15:**
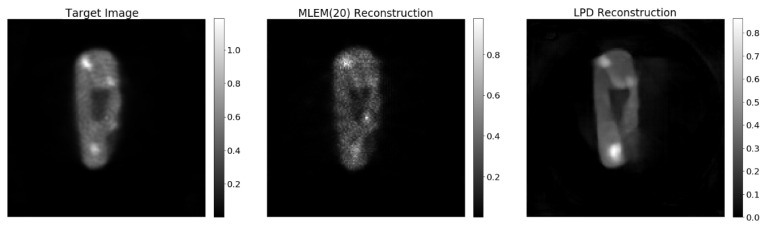
Target image, ${\mathrm{MLEM}}_{20}$, LPD reconstruction on 40% of projection data.

The LPD-algorithm, by design, switches back and forth between data and image space and is forced to compare the forward projection of each iterate with the data at hand. Even though this gives some hope for robustness of the LPD-algorithm against variation of the data, it is still so that the neural networks in the LPD-algorithm perform best when the noise level of the data to reconstruct is well represented in the training set. This can be appreciated by looking at Figure 15, where the LPD-algorithm has been applied to only 40% of the data. While the main features of the object are still captured and the cold-area-to-background contrast is well preserved, the activity concentration is underestimated and shape artefacts appear in low concentration regions. There are different ways to solve such shortcomings. The number of iterations of the LPD-algorithm could be increased, giving rise to a model with more parameters that could be able to better handle discrepancy between training set and data to reconstruct; the training set could be enlarged as to accommodate a larger spectrum of data sets; or, most probably, both of the above should be attempted in synergy. Each of those attempts requires extensive training and tests, and we were not able to investigate any of those in the present work.

On the bright side, the LPD-algorithm seems to be able to reconstruct objects of shapes that can be very different from the ones present in the training set, probably due to the fact that projections data are given as input at each iteration stage, enforcing data fidelity.

A small comment needs to be made about the choice for experimental training data. The fact that we chose to create 60 data sets with 60 different noise levels by adding up 60 min of acquisition, one minute at the time, makes the sinograms in our training set correlated. Our opinion is that this does not constitute a problem for our purpose, but it can be argued that we are overestimating the size of our training sets.

## 5. Conclusions

The Learned Primal Dual algorithm introduced by Adler and Öktem [12] has been used to reconstruct PET data from a small animal imaging scanner (miniPET 3). The algorithm has been trained with an hybrid data set consisting of a large number of synthetic and a relatively small number of experimental data. The results are encouraging with good recovery of the activity concentrations, fair reconstruction of the shapes in the test object and a substantial noise reduction. No introduction of artefacts when reconstructing objects quite different from the ones used for training has been observed. Another aspect worth noting is that, once trained, the LPD-algorithm gives a reconstruction in fractions of a second (or few seconds if the data loading is taken into account), which is substantially less than the few minutes needed for a standard iterative reconstruction of similar quality.

## Figures and Tables

**Table 1:** Fludeoxyglucose (FDG)activity concentrations for the various measurements with training phantoms. The letter on the top row corresponds to the volume indicated in Figure 4 and Figure 5 for the respective phantom.

		A[$\frac{\mathrm{MBq}}{\mathrm{mL}}$]	B[$\frac{\mathrm{MBq}}{\mathrm{mL}}$]	C[$\frac{\mathrm{MBq}}{\mathrm{mL}}$]	D[$\frac{\mathrm{MBq}}{\mathrm{mL}}$]	E[$\frac{\mathrm{MBq}}{\mathrm{mL}}$]
1	M1	3.1	2.3	3.1	0.2	-
	M2	1.0	2.5	1.0	0.1	-
	M3	2.0	0.8	0.8	0.3	-
	M4	0	0	0	0.3	-
	M5	2.1	2.9	2.9	0.5	-
	M6	1.3	1.7	1.7	0.2	-
2	M1	1.5	0.3	0.3	0.08	-
	M2	0.9	0.6	0.6	0.2	-
	M3	1.2	0.5	1.2	0.2	-
	M4	0	0	0	0.1	-
	M5	0	0	0	0.2	-
3	M1	1.3	2.7	1.3	0.09	0
	M2	1.1	0.4	1.1	0.07	0
	M3	0.5	0.2	0.5	0.05	0
	M4	0	0	0	0.2	0
	M5	1.4	0.9	0.9	0.2	0
	M6	0.7	0.4	0.4	0.1	0.3

**Table 2:** Activity concentrations for the various measurements with test phantom.

		Body[$\frac{\mathrm{MBq}}{\mathrm{mL}}$]	Brain[$\frac{\mathrm{MBq}}{\mathrm{mL}}$]	Heart[$\frac{\mathrm{MBq}}{\mathrm{mL}}$]	Lungs[$\frac{\mathrm{MBq}}{\mathrm{mL}}$]	Kidneys[$\frac{\mathrm{MBq}}{\mathrm{mL}}$]	Bladder[$\frac{\mathrm{MBq}}{\mathrm{mL}}$]
T	M1	0.5	1.1	0.1	0.15	0.8	1.3
	M2	0.4	1.1	0.1	0.07	0.9	out of FOV

**Table 3:** PSNR and SSIM for 3-iteration LPD- and MLEM-algorithm on synthetic data.

	PSNR	ΔPSNR	SSIM	ΔSSIM
LPD	24.36	3.98	0.87	0.17
MLEM	20.38	-	0.70	-

**Table 4:** PSNR and SSIM for LPD-algorithm on miniPET data, evaluated against the target reconstruction. ΔPSNR and ΔSSIM are computed by subtracting the respective value of 20-iterations MLEM on the same data.

	LPD 3 Iterations miniPET
PSNR	25.20
ΔPSNR	+2.59
SSIM	0.60
ΔSSIM	−0.18

## Data Availability

Not applicable.
